# Successful treatment with mesenchymal stromal cells-Frankfurt am Main in a pediatric patient with steroid-refractory and ruxolitinib-refractory acute gastrointestinal graft-versus-host disease

**DOI:** 10.1007/s00277-025-06491-y

**Published:** 2025-07-15

**Authors:** Jana Ernst, Steffen Reinsch, Hans-Joachim Mentzel, Till Milde, Bernd Gruhn

**Affiliations:** 1 Department of Pediatric and Adolescent Medicine, Jena University Hospital, Jena, Germany; 2 Comprehensive Cancer Center Central Germany (CCCG), Jena, Germany; 3 Section of Pediatric Radiology, Department of Radiology, Jena University Hospital, Jena, Germany; 4 Hopp Children’s Cancer Center Heidelberg (KiTZ), Heidelberg, Germany; 5 Clinical Cooperation Unit Pediatric Oncology, German Cancer Research Center Heidelberg (DKFZ), Heidelberg, Germany

**Keywords:** Hematopoietic stem cell transplantation, Acute graft-versus-host disease, Mesenchymal stromal cells, MSC-FFM, Calprotectin, Intestinal wall thickness

## Abstract

Acute graft-versus-host disease (aGVHD) as complication after allogeneic hematopoetic stem cell transplantation (allo-HSCT) occurs in up to 70% of patients. The survival prognosis depends on the severity of GVHD and response to first-line therapy. In patients who are refractory to both, steroids and ruxolitinib, an unmet medical need exists, especially in severe gastrointestinal forms of aGVHD. We report the case of a 12-year-old male patient who developed severe gastrointestinal aGVHD after allo-HSCT for the treatment of high-risk acute myeloid leukemia. The grade IV aGVHD of the intestine showed no improvement under triple immunosuppression with corticosteroids, cyclosporine A and ruxolitinib. After receiving four weekly infusions of human allogeneic mesenchymal stromal cells DRK-BaWü-He-FFM (MSC-FFM), intestinal inflammation finally improved, as reflected by ameliorated symptoms, normalized intestinal wall thickness (ultrasonography) and decreasing calprotectin levels. Intestinal mucosal healing was further supported by a strict, formula-based modular diet. The present case supports efficacy and safety of MSC-FFM in combination with modular nutrition in complicated courses of aGVHD of the intestine refractory to multiple lines of therapy and introduces intestinal wall thickness and calprotectin levels as valuable markers of disease activity.

## Introduction

Graft-versus-host disease (GVHD) is a multisystemic, inflammatory and/or fibrotic disease significantly contributing to morbidity and non-relapse mortality after allogeneic hematopoietic stem cell transplantation (allo-HSCT). Classic acute GVHD (aGVHD) is a reaction of transplanted donor immune effector cells against epithelial structures of the host, occurring within 100 days after transplantation. Targeted organs include the skin, the lower and upper gastrointestinal tract and the liver, resulting in typical signs and symptoms such as erythema, maculopapular rash, nausea, vomiting, anorexia, profuse diarrhea, ileus, or cholestatic liver disease [1]. Acute GVHD is one of the most frequent complications of allo-HSCT and is associated with delayed recovery and complicated post-transplant courses, particularly in patients who do not respond to first-line treatment with steroids [2]. The risk of developing GVHD is dependent on various factors, mainly on the degree of match between donor and recipient, graft type, T cell depletion/repletion, conditioning regimen, GVHD prophylaxis, and age of the patient/donor [3–5].

Although incidence and mortality of aGVHD have decreased during recent years, aGVHD as a post-transplant complication still occurs in up to 70% of allo-HSCT patients [6]. The severity of aGVHD has a major impact on survival prognosis, and clinically significant grades II to IV remain challenging as one of the leading causes of non-relapse mortality in patients undergoing allo-HSCT. Another important factor for morbidity and mortality is the response to first-line therapy, with approximately 50% of aGVHD patients responding to high-dose corticosteroids. However, the response rate in patients with grade IV disease does not exceed 30% [7] and the outcome for patients who do not respond to corticosteroids (steroid-resistant aGVHD [SR-aGVHD]) is poor, with long-term survival ranging from 5 to 30% [8]. In patients with gastrointestinal forms of the disease, the percentage of SR-aGVHD is even higher, and they are particularly vulnerable to life-threatening complications such as infections, bleeding, and surgical complications, resulting in a median survival of less than 12 months [7].

Though the definition of SR-aGVHD is somewhat heterogeneous across studies and publications, some agreement has been reached in defining corticosteroid-refractory aGVHD as.

- disease progression after 3 days of treatment with methylprednisolone 2 mg/kg per day equivalent,
- lack of improvement after 7 d of treatment with methylprednisolone 2 mg/kg per day equivalent,
- progression to a new organ after treatment with methylprednisolone 1 mg/kg per day equivalent for skin and upper gastrointestinal GVHD, or
- recurrence during or after a corticosteroid taper [9].

No approved therapeutic options are available for gastrointestinal aGVHD (GI-aGVHD) that is refractory to both, first-line treatment with steroids and second-line treatment with the Janus Kinase 1/2 (JAK1/2) inhibitor ruxolitinib (ruxolitinib-refractory aGVHD [RR-aGVHD]), and therefore RR-aGVHD continues to pose a high unmet medical need [10]. We report the case of a 12-year-old boy with severe gastrointestinal (GI)-RR-aGVHD who was successfully treated with a human allogeneic mesenchymal stromal cell product (allogeneic mesenchymal stromal cells DRK-BaWü-He-FFM [MSC-FFM], OBNITIX^®^, medac Gesellschaft für klinische Spezialpräparate mbH, Wedel, Germany) complemented by modular nutrition (Modulen IBD, MODULEN^®^, Nestlé Health Science GmbH, Frankfurt am Main, Germany).

## Case presentation

A 12-year-old patient with high-risk acute myeloid leukemia (AML; FAB classification M0, translocation t(6;11) with KMT2A::MLLT4 fusion) in complete molecular remission after chemotherapy according to the AML-BFM 2019 protocol, was admitted for allo-HSCT to the Stem Cell Transplantation Unit, Department of Pediatrics, Jena University Hospital, in May 2023. The patient was in good general and nutritional condition (body weight [BW] 35 kg) and had no relevant co-morbidities. Bone marrow examination on day − 11 before allo-HSCT showed complete remission (minimal residual disease [MRD] negative).

Allogeneic human peripheral blood stem cells from a matched unrelated donor (10/10 HLA-matched, male) were transplanted on 2 consecutive days in late June 2023 (divided into 2 doses based on the maximum possible daily Dimethyl sulfoxide [DSMO] dose) after conditioning according to the AML-SZT-BFM 2020 protocol (EudraCT number: 2020-005634-15, busulfan/cyclophosphamide/melphalan). GVHD prophylaxis was applied according to protocol, with antithymocyte globulin from day − 3 to −1 and cyclosporine A from day − 3 until discharge.

From day + 13 the patient showed an engraftment syndrome with a rapid increase in leukocytes and neutrophils, a generalized exanthema, bone pain and fever, that was treated with methylprednisolone and topical pimecrolimus, which led to a rapid and complete improvement. Engraftment was confirmed by an absolute neutrophil count (ANC) > 0.5 Gpt/L in peripheral blood and evidence of all 3 cell lines, without detecting leukemia cells in cytologic bone marrow examinations. Good graft function was also demonstrated by a 100% chimerism from day + 27 and beyond. MRD remained negative until discharge.

**Fig. 1:**
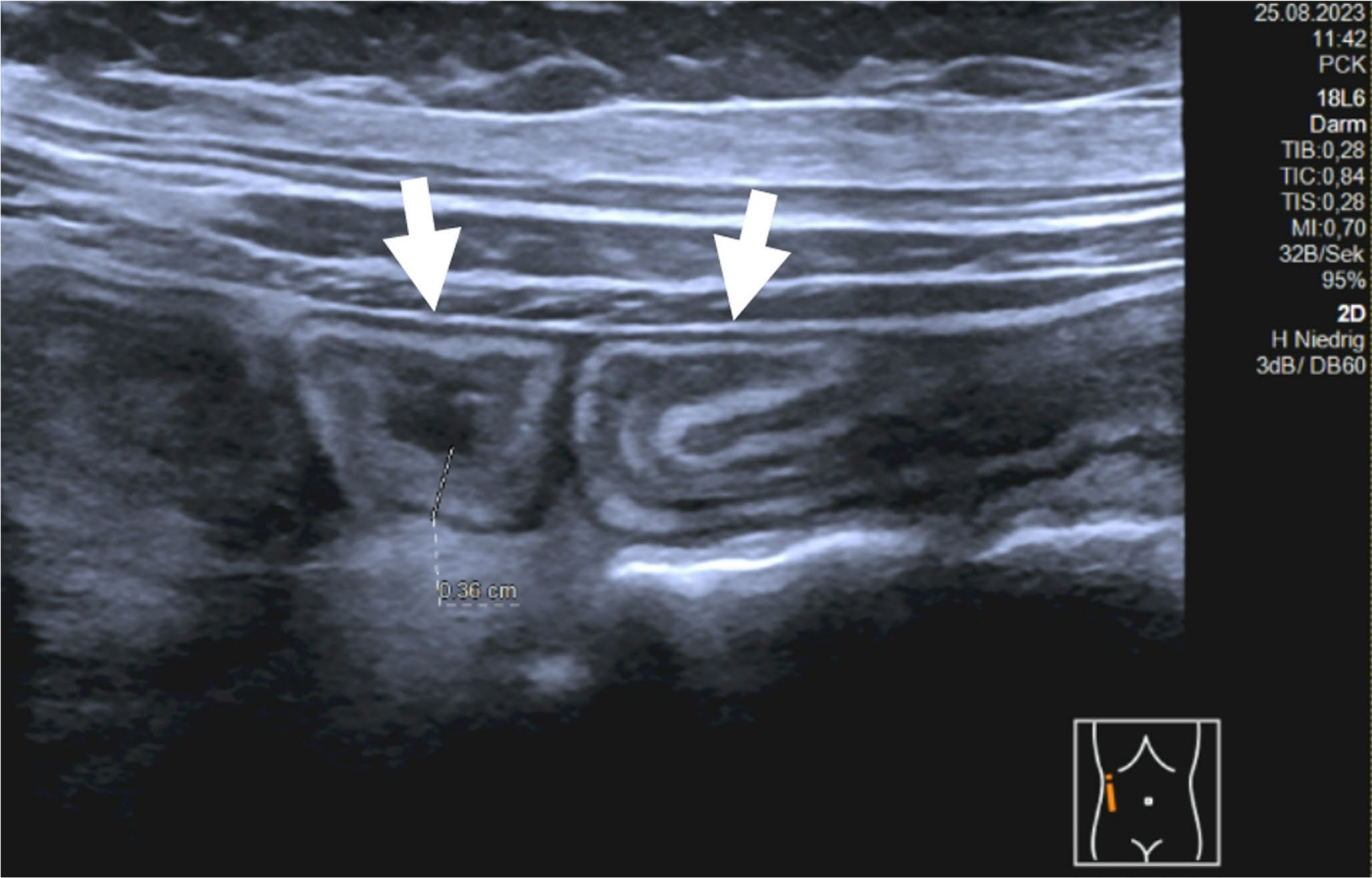
B-mode image showing blurred bowel and thickened wall structure (arrows) in the right middle abdomen

From day + 26, the patient developed diarrhea accompanied by increasing severe abdominal pain, and transabdominal ultrasonography (day + 46) showed a generalized thickening of the intestinal wall by nearly 4 mm, corresponding to GI-aGVHD (Fig. 1). Histopathology of gastro-colonoscopy biopsies indicated aGVHD grade II to III in the terminal ileum and duodenum, plus grade I to focal II lesions in the colorectum. The clinical picture in combination with the diagnostic findings were consistent with the diagnosis of grade IV aGVHD [11].

First-line aGVHD treatment with methylprednisolone at the standard dose of 2 mg/kgBW/d was initiated from day + 47, resulting in a subtle improvement of symptoms, accompanied by a decrease in bowel wall thickness on sonography. However, methylprednisolone could not be tapered and the patient steadily lost weight (> 5 kg, i.e. >15% BW). Therefore, additional immunosuppressive therapy with ruxolitinib was initiated. However, even under triple immunosuppression (including cyclosporine A from day − 3,), the clinical situation deteriorated further with increasingly thin stools (1500 mL/d, i.e. >30 mL/kg/d), metabolic acidosis, high protein loss and a significant increase in stool calprotectin as an inflammatory marker (up to max. 978 µg/g stool).

Ultrasonography showed persistent intestinal wall thickening, confirming (re-)progression to grade IV GI-RR-aGVHD, and therapy with mesenchymal stromal cells (MSC-FFM) was initiated on day + 70. Following premedication with dimetindene, four weekly infusions (1–2 million cells/kg BW per dose) were administered. All four doses were well tolerated, no acute or late toxicities were observed. Concurrently, a special drinking diet, usually indicated for the treatment of Crohn’s disease (Modulen IBD), was given to relieve the GI tract and to maintain enteral nutrition.

**Fig. 2:**
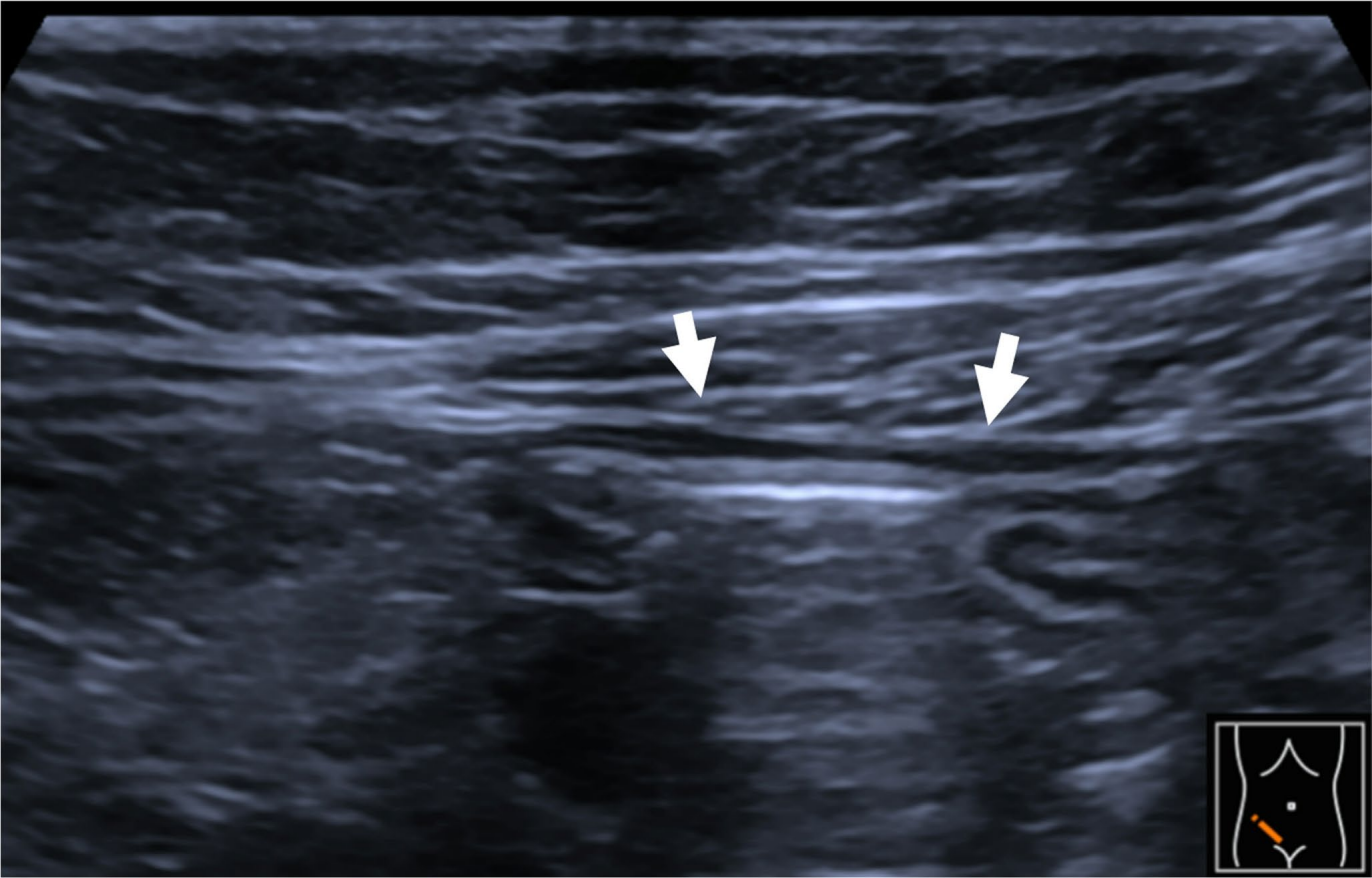
B-mode image showing normalized bowel wall structure (arrows) in the right middle abdomen

**Fig. 3:**
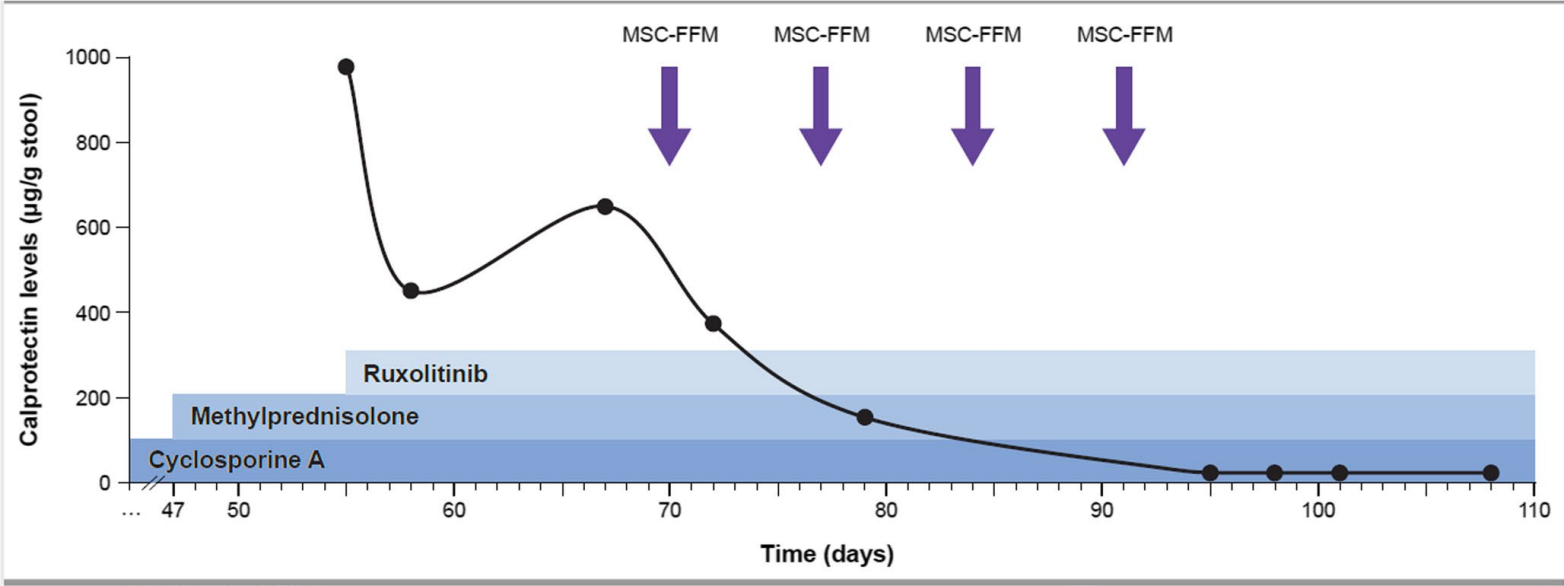
Calprotectin levels significantly decreased during treatment with MSC-FFM

With this management, including triple immunosuppressive chemotherapy plus full dose MSC-FFM plus the Modulen IBD liquid diet, the patient showed slow but steady symptom relief, especially diarrhea (stool volume and frequency decreased), metabolic acidosis and protein loss were corrected. In parallel, sonography findings improved, showing a well-structured bowel wall without signs of thickening (Fig. 2), and stool calprotectin levels returned to normal (< 27 µg/g stool) by day + 95 (Fig. 3). Methylprednisolone was tapered and finally discontinued. Exclusive enteral nutrition has been continued for six weeks and then changed to a combination with western diet and enteral nutrition. The patient was discharged in good general condition with a discharge weight of 32.7 kg on day + 101. At 22 months post-allo-HSCT, the patient is doing well and in molecular remission of AML.

## Discussion

Acute GVHD remains a major challenge in the management of allo-HSCT, particularly for patients with severe SR-aGVHD. The long-term survival rates are alarmingly low, with a 4-year survival of approximately 15%. However, mortality is not only due to aGVHD itself, but also due to adverse effects of treatment with immunosuppressants, as these predispose to opportunistic infections and the loss of the graft-versus-leukemia effect [9–13]. Not surprisingly, infections are still the leading cause of non-relapse mortality in patients after allo-HSCT, followed by organ failure, while SR-aGVHD is considered the third leading cause of death in patients with predominant GI manifestations [7].

A second-line treatment for aGVHD is recommended if corticosteroid resistance or dependence occurs. In May 2022, ruxolitinib (JAKAVI^®^) was approved for the treatment of patients aged 12 years and older with acute or chronic GVHD who have inadequate response to corticosteroids or other systemic therapies. The approval of ruxolitinib in aGVHD was based on the Phase III REACH II which showed a 62% overall response rate (ORR) with ruxolitinib at Day 28, compared to 39% for best available therapy (BAT) [14, 15]. Following these results and the subsequent approval, ruxolitinib has since become the new standard of care in SR-aGVHD.

The recently published REACH 4 study confirmed the efficacy and safety of ruxolitinib also in pediatric patients with treatment-naive and steroid-refractory grade II-IV aGVHD (*n* = 45). This phase I/II study showed an ORR of 84.4% in all patients (90% confidence interval [CI], 72.8–92.5) at day 28, with a durable ORR of 66.7% (90% CI, 53.4–78.2) at day 56 [16]. Data about other novel agents used in the pediatric population of aGvHD patients is scarce. Teduglutide, a human recombinant GLP-2 analog, may be beneficial as an adjunctive and non-immune suppressive agent for managing GI-aGVHD, due to its tissue protective and regenerative properties [17]. Vedolizumab, a novel anti-α4β7 integrin monoclonal antibody, has shown promising activity for the treatment of lower GI aGVHD, but caution for concurrent infections is warranted [18]. However, these agents need to be evaluated in larger prospective clinical trials in order to establish their efficacy and safety to treat pediatric aGVHD patients.

With regards to the pivotal data for ruxolitinib, both studies show that there is a significant number of patients who do not respond adequately to therapy with ruxolitinib or who do not tolerate it, and further treatment options are urgently needed. The unfavourable prognosis for patients who fail both, steroids and ruxolitinib, was demonstrated by an analysis of adult patients with severe RR-aGVHD, showing a median survival of only 28 days (interquartile range [IQR]: 7–90 days) [19].

Mesenchymal stromal cells are multipotent progenitor cells, which exist in most human tissues. They can be isolated and expanded ex vivo from bone marrow, umbilical cord blood, adipose tissue, and placenta. Regardless of the tissue of origin, all cells called “MSCs” are spindle-shaped, express the cluster of differentiation (CD) markers CD73, CD90, and CD105, lack expression of hematopoietic stem cell markers, and can differentiate into chondroblasts, osteoblasts, and adipocytes under appropriate culture conditions [20]. Their powerful regenerative and immunomodulatory capacities combined with low immunogenicity make these cells attractive candidates for the treatment of aGVHD.

MSC-FFM, derived from a mononuclear bone marrow cell pool of at least 8 healthy donors, minimizes donor-to-donor heterogeneity, which may lead to inconsistent results after single donor MSC treatments [12, 21]. In a proof-of-concept study with 69 patients (51 children and 18 adults), *Bader et al.* showed that MSC-FFM is an effective therapy for SR-aGVHD: the ORR after 28 days was 83%; at the last follow-up, 61% and 25% of patients were in complete or partial remission, respectively. This was associated with a higher probability of 6-month overall survival of 71% (range 61–83%) compared to the results of patients not treated with MSC-FFM [13].

Furthermore, recent real-world data suggest that RR-aGVHD patients may also benefit from MSC-FFM treatment. The retrospective analysis included 156 patients, 33 of whom were children, with a range from 0 to 17 years (median 9 years; IQR 6–12 years). These children were heavily pretreated, with a median number of 7 previous aGVHD therapies (range 2–11) including ruxolitinib, but most still suffered from aGVHD grade III/IV (30% grade III, 64% grade IV) [12].

With MSC-FFM, the ORR at day 28 was 64% in pediatric RR-aGVHD patients, with mostly durable responses [11]. The probability of overall survival (OS) at 6, 12 and 24 months was 59%, 42% and 35%, respectively, which compares favorably to OS estimates of 20% at 6 months, 16% at 12 months, and 10% at 24 months for adult RR-aGVHD patients receiving BAT after ruxolitinib failure [19]. In this adult patient population, receiving BAT after ruxolitinib, median OS was 28 days, compared to 5.8 months observed in the overall patient population treated with MSC-FFM [12, 19]. The safety profile of MSC-FFM was consistent with the data available to date, and no adverse drug reactions were reported in pediatric RR-aGVHD patients. Of note, MSCs do not suppress hematopoiesis or adaptive immune responses; thus, the incidence of hematological or infectious (e.g. re-activations of latent viral infections) complications is low [12].

The presented case provides additional evidence for MSC-FFM’s potential in treating children with severe SR-aGVHD not adequately responding to ruxolitinib, which is also reflected by the development of the intestinal wall thickness, measured by ultrasonography: the more active the aGVHD, the thicker was the intestinal wall. Furthermore, blood flow was increased in the inflamed, thickened intestine, while values returned to normal after 4 doses of MSC-FFM (Figs. 1 and 2). Sonography is a non-invasive and easily accessible diagnostic tool, which is especially valuable for the management of pediatric aGvHD patients.

Another non-invasive measure to evaluate inflammatory disease activity in the intestine - and to monitor treatment success - was the course of calprotectin levels in stool. Calprotectin, a neutrophil-derived protein, is markedly elevated in stool and/or plasma in infectious and inflammatory conditions, and elevated concentrations of fecal calprotectin have been detected in patients with inflammatory bowel disease (IBD). In this patient population, it has been demonstrated, that increased calprotectin levels in stool correlate well with histological inflammation detected by colonoscopy with biopsies [22]. In our patient, amelioration of aGVHD symptoms was accompanied by decreasing calprotectin levels, whereas deterioration was followed by an increase in fecal calprotectin. Hence, the fluctuating course of the calprotectin levels in stool mirrored the clinical course of the patient, indicating that this might be a valuable clinical marker for measuring disease activity in GI-aGVHD. This is also supported by review of the literature, indicating that median fecal calprotectin levels can be used for diagnosing as well as predicting the treatment response for GI-aGVHD. However, future randomized prospective trials involving larger populations are needed to further explore its significance [23].

As a noninvasive measure, it is patient-friendly and convenient in clinical practice, especially in children. Furthermore, as a parameter measured from one stool sample per day it is less time-consuming and more feasible than measuring the entire stool volume of a day, which is usually used for the staging of GVHD. Measurement of stool volume per day is a difficult procedure to perform, as stool and urine often cannot be separated, especially in children, resulting in falsely increased or decreased stool volume values.

Even though nutrition is essential for the management of children with aGVHD, currently, no consensus exists for the nutritional management of these patients [24]. However, nutrition also played an important role in this patient’s recovery. In addition to the immunosuppressive triplet therapy and the immunomodulatory treatment with MSC-FFM, the patient was solely fed the elementary diet Modulen IBD when aGVHD was highly active. Modulen IBD is used as a first-line treatment for the IBD Crohn’s Disease as exclusive enteral nutrition and consists of easily digestible carbohydrates and medium chain fatty acids, high-quality milk protein and the anti-inflammatory transforming growth factor β2 (TGF-β2). It reduces inflammation in the intestine and helps the intestinal villi, often damaged by GVHD, to recover and has been shown to change the microbiom in these patients. In contrast to immunosuppressive therapy, therapy with exclusive enteral nutrition (Modulen) has no side effects, therefore, it is an integral part of therapy-refractory GI-aGVHD management in our hospital.

In summary, this case demonstrates that.

- MSC-FFM have the potential to treat pediatric patients with SR-aGVHD effectively and safely, even after ruxolitinib failure,
- measurement of intestinal wall thickness (ultrasonography) and fecal calprotectin levels are valuable diagnostic tools to monitor GI-aGVHD disease activity and response to treatment, and
- nutrition with Modulen IBD contributes significantly to aGVHD therapy, as it promotes intestinal healing without the risk for adverse reactions and maintains enteral feeding to avoid parenteral nutrition.

## Data Availability

No datasets were generated or analysed during the current study.
